# Involvement of Different CD4^+^ T Cell Subsets Producing Granzyme B in the Immune Response to *Leishmania major* Antigens

**DOI:** 10.1155/2014/636039

**Published:** 2014-07-02

**Authors:** Ikbel Naouar, Thouraya Boussoffara, Melika Ben Ahmed, Nabil Belhaj Hmida, Adel Gharbi, Sami Gritli, Afif Ben Salah, Hechmi Louzir

**Affiliations:** ^1^Laboratory of Transmission, Control, and Immunobiology of Infections-LR11IPT02, Pasteur Institute of Tunis, 13 Place Pasteur, 1002 Tunis, Tunisia; ^2^University of Tunis El Manar, 1068 Tunis, Tunisia; ^3^Department of Pathology, Charles Nicolle Hospital, Boulevard 9 Avril 1938, 1006 Tunis, Tunisia

## Abstract

The nature of effector cells and the potential immunogenicity of *Leishmania major* excreted/secreted proteins (*Lm*ES) were evaluated using peripheral blood mononuclear cells (PBMCs) from healed zoonotic cutaneous leishmaniasis individuals (HZCL) and healthy controls (HC). First, we found that PBMCs from HZCL individuals proliferate and produce high levels of IFN-*γ* and granzyme B (GrB), used as a marker of activated cytotoxic T cells, in response to the parasite antigens. IFN-*γ* is produced by CD4^+^ T cells, but unexpectedly GrB is also produced by CD4^+^ T cells in response to stimulation with *Lm*ES, which were found to be as effective as soluble *Leishmania* antigens to induce proliferation and cytokine production by PBMCs from immune individuals. To address the question of regulatory T cell (Tregs) involvement, the frequency of circulating Tregs was assessed and found to be higher in HZCL individuals compared to that of HC. Furthermore, both CD4^+^CD25^+^ and CD4^+^CD25^−^ T cells, purified from HZCL individuals, produced IFN-*γ* and GrB when stimulated with *Lm*ES. Additional experiments showed that CD4^+^CD25^+^CD127^dim/−^ Tregs were involved in GrB production. Collectively, our data indicate that *Lm*ES are immunogenic in humans and emphasize the involvement of CD4^+^ T cells including activated and regulatory T cells in the immune response against parasite antigens.

## 1. Introduction

Cutaneous leishmaniasis is endemic in the tropics and neotropics. It is often referred to as a group of diseases because of the varied spectrum of clinical manifestations, which range from small cutaneous nodules to gross mucosal tissue destruction. Cutaneous leishmaniasis can be caused by several* Leishmania* spp and is transmitted to humans and animals by sandflies [1].

Zoonotic cutaneous leishmaniasis (ZCL), a highly prevalent disease in North Africa, sub-Saharan West Africa, Middle East, and Central Asia, is caused by* Leishmania major* [2]. In humans, previous studies have reported that healing of cutaneous leishmaniasis is generally associated with the development of a cellular immune response against the parasite, as well as a positive leishmanin skin test (LST) reactivity [1, 3]. Moreover, healing is usually correlated with resistance to a subsequent symptomatic infection [4, 5] demonstrating that immunity against leishmaniasis is possible and can be achieved through vaccination. The prevailing view is that Th1 responses are essential for the control of parasite multiplication. Previously, we have demonstrated that parasite-specific cytotoxic immune responses are developed by individuals living in areas of* L. major* transmission and could play a crucial role in resistance to reinfection [6, 7]. Therefore, it seems that elucidation of the specific effective immunological mechanisms and the cell populations operating in the resistance against human* Leishmania* infection is fundamental for vaccine development.

In this setting, we were interested in the excreted/secreted proteins of* Leishmania major*. Indeed, it has previously been hypothesized that the secreted and surface molecules are mainly important for the establishment of infection, protecting the parasite from the early action of the host immune system and acting as invasive/evasive determinants [8]. In addition, it was reported in animal models that excreted/secreted molecules from other intracellular pathogens, such as* Mycobacterium tuberculosis* and* Toxoplasma gondii,* contain highly immunogenic and protective antigens [9–11]. Furthermore, there is evidence that* Leishmania* promastigote culture filtrate proteins eliciting a strong protective immunity against the infection in BALB/c mice [12–14]. Similarly, in dogs,* L. infantum* excreted/secreted antigens inducing a long lasting and strong immune response against canine visceral leishmaniasis [15–17].

In the present study, we aimed at better evaluating the nature of the cellular effectors involved in* Leishmania* infection by focusing on the cytotoxic immune response induced by the parasite antigens, and at validating* Lm*ES as potential target of this immune response.

## 2. Materials and Methods

### 2.1. Study Population and Samples

Peripheral blood samples were obtained from 15 individuals living in Sidi Bouzid Governorate (Central Tunisia); an endemic area of* L. major* infection. These HZCL individuals had characteristic scars of leishmaniasis on skin examination and/or a positive LST (mean induration > 5 mm) and/or a positive lymphoproliferative response to soluble* Leishmania* antigens (SLA) (Table 1). Thirteen healthy individuals living in Tunis, a nonendemic region for ZCL, were included as healthy controls (HC), (Table 1). All subjects provided written informed consent for participation in the study and sample collection and analyses. The protocol was approved by the Institutional Review Board of the Pasteur Institute of Tunis.

### 2.2. Leishmania major Excreted/Secreted Proteins and Soluble Leishmania Antigen Preparation

*L. major *parasites (MHOM/TN/94/GLC94, zymodeme MON25) were cultured on Novy-Nicolle-McNeal medium at 26°C and progressively adapted to RPMI 1640 medium (Sigma, St. Louis, MO) containing 2 mM L-glutamine (Sigma, St. Louis, MO), 100 U/mL penicillin (Sigma, St. Louis, MO), 100 mg/mL streptomycin (Sigma, St. Louis, MO), and 10% heat-inactivated fetal calf serum (FCS) (Invitrogen, Cergy-Pontoise, France). Stationary-phase promastigotes were used for preparation of SLA as previously described [18]. Preparation of* Lm*ES was performed as described by Chenik and collaborators [19]. Proteins were analyzed by SDS-PAGE and their concentrations were determined with a Bradford protein assay.

### 2.3. Leishmanin Skin Test

LST was performed by intradermal injection of 100 *μ*L of leishmanin suspension containing 5 × 10^6^ *L. major* promastigotes/mL in 0.5% phenol saline. After 72 h, the induration was measured along 2 diameters by the ballpoint pen technique. Induration with a diameter of 5 mm or more indicated a positive test.

### 2.4. Isolation of Human PBMCs and T Cell Subsets

PBMCs were separated from heparinized blood samples using Ficoll-Paque (GE Healthcare, Uppsala, Sweden) density gradient centrifugation. In some experiments, CD8^+^ or CD4^+^ T cells were depleted directly from PBMCs according to the manufacturer's recommendations (Miltenyi Biotec GmbH, Bergisch Gladbach, Germany). CD4^+^CD25^+^ T-lymphocytes were purified by a two-step immunomagnetic technique: purification of CD4^+^ T-lymphocytes using a mAb-cocktail (Miltenyi Biotec GmbH, Bergisch Gladbach, Germany) and positive selection of CD4^+^CD25^+^ T-lymphocytes using anti-CD25 magnetic microbeads.

The isolation of CD4^+^CD25^+^CD127^dim/−^ regulatory T cells was also performed in a two-step procedure. First, the non-CD4^+^ and CD127^high^ cells are indirectly magnetically labeled with a cocktail of biotin-conjugated antibodies. In the second step, the CD4^+^CD25^+^CD127^dim/−^ regulatory T cells are directly labeled with CD25 MicroBeads and isolated by positive selection from the preenriched CD4^+^ T cell fraction. The purity of each depleted fraction was assessed by flow cytometry and was above 85% for all samples.

### 2.5. Lymphoproliferative Tests

Total or depleted PBMCs were cultured in 96-well plates at a concentration of 1 × 10^6^ cells/mL in a final volume of 200 *μ*L of complete medium containing RPMI 1640 medium (Sigma, St. Louis, MO) supplemented with 2 mM L*-*glutamine (Sigma, St. Louis, MO), 100 U/mL penicillin (Sigma, St. Louis, MO), 100 *μ*g/mL streptomycin (Sigma, St. Louis, MO), and 10% (v/v) heat-inactivated human AB serum (Sigma, St. Louis, MO), 1% HEPES (0.01 M), (Invitrogen, Cergy-Pontoise, France), 1% sodium pyruvate (1 mM) (Invitrogen, Cergy-Pontoise, France), 1% MEM Non-Essential Amino Acids (Invitrogen, Cergy-Pontoise, France), 1‰ 2-Mercaptoethanol (10^−2 ^M), (Invitrogen, Cergy-Pontoise, France), and 0.2% gentamicin (20 *μ*g/mL) (Invitrogen, Cergy-Pontoise, France). PBMCs were stimulated with* Lm*ES (10 *μ*g/mL) or SLA (10 *μ*g/mL) for five days. The uptake of [^3^H]-thymidine (Amersham, Saclay, France) was measured after adding 1 mCi/well for the last 6 h and evaluated for cell proliferation in a liquid scintillation counter (Rack Beta, LKB Wallace, Australia). Results were expressed as a proliferation index: mean counts of triplicates in antigen-stimulated cultures/mean counts of triplicates in unstimulated cultures.

### 2.6. Antigen-Presenting Cell Preparation and Co-Culture with Effector Cells

PBMCs were treated with 50 *μ*g/mL mitomycin C (Sigma, St. Louis, MO) at 2 × 10^6^ cells/mL in RPMI supplemented with 10% FCS for 1 h at 37°C followed by 3 washes with RPMI 1640 medium. These cells were used as antigen-presenting cells (APCs**)** and incubated in the presence of* Lm*ES (10 *μ*g/mL), alone or with CD4^+^CD25^+^, CD4^+^CD25^−^, or CD4^+^CD25^+^CD127^dim/−^ T cells at different ratio of effector cells/APCs (5/1 to 50/1). The number of APCs was kept constant and the number of effector cells was increasing according to the appropriate ratios. Supernatants were collected after 5 days of culture and stored at −80°C until use.

### 2.7. GrB and IFN-*γ* Detection in Culture Supernatants

Measurement of GrB was carried out using commercially available Human Granzyme B ELISA set (Mabtech AB, Nacka Strand, Sweden) according to the manufacturer's recommendations. OptEIA set ELISA Set (BD Biosciences, San Jose, CA) was used to detect IFN-*γ*. The results were interpolated from a standard curve using recombinant cytokines and expressed in pg/mL.

### 2.8. Flow Cytometry Analyses

PBMCs (2 × 10^6^ cells/well) were stimulated* in vitro* for 16 h in presence of* Lm*ES (10 *μ*g/mL), or medium alone with Golgistop (BD Biosciences, San Jose, CA) added for the last 6 h of culture. Cells were surface stained (with PerCP-CD3, PE-Cy7-CD4, anti-CD28, anti-CD25, anti-CD57, and anti-CD16 coupled to FITC), fixed, and permeabilized using BD Cytoperm/cytofix plus kit (BD Biosciences, San Jose, CA) according to the manufacturer's recommendations and stained with PE-conjugated anti-human GrB (Fitzgerald Industries Inc., BioLegend, London, UK) and APC-conjugated anti-human IFN-*γ* (BD Biosciences, San Jose, CA). A total of 100,000 events were acquired for all samples. Analyses were performed with a FACS Canto flow cytometer using the FACS Diva software (BD Biosciences, San Jose, CA).

### 2.9. ELISpot Assay Specific for GrB and IFN-*γ*

The dual ELISpot assay specific for GrB and IFN-*γ* was performed to quantify* Lm*ES-specific T cells. In brief, PBMCs or purified CD4^+^ and CD8^+^ T cells were cultured at room temperature for 30 min in the presence or absence of* Lm*ES at a final concentration of 10 *μ*g/mL. For positive controls, cells were stimulated with 10 *μ*g/mL phytohaemagglutinin (Sigma, St. Louis, MO). Cells under stimulation were then transferred in a PVDF-bottomed-well ELISpot plate coated with IFN-*γ* and GrB capture antibodies (Abcam, Cambridge, MA) and incubated overnight in a humidified 5% CO_2_ incubator at 37°C. The plates were further washed and incubated with anti-FITC HRP and streptavidin-alkaline phosphatase conjugates for 1 h at 37°C. The plates were then developed, first using AEC buffer and then BCIP/NBT buffer. Spots were analyzed using CTL ImmunoSpot reader (CTL Analyzers, Shaker Hights, OH). Data were expressed as the mean spot-forming units (SFU) per 10^6^ cells calculated after subtracting spots of the negative controls.

### 2.10. Statistical Analyses

Mann-Whitney test was used for comparison of lymphoproliferative response and induction of GrB and IFN-*γ* between the different study groups. Correlation between GrB and IFN-*γ* levels was estimated using Spearman's rank order correlation coefficient. Statistical analyses were performed using SPSS 10.0 statistical program (IBM, Armork, NY). Statistical significance was assigned to a value of *P* < 0.05.

## 3. Results

### 3.1. A Strong Correlation Is Observed Between IFN-*γ* and GrB Production in Response to Leishmania Antigens

**Figure 1:**
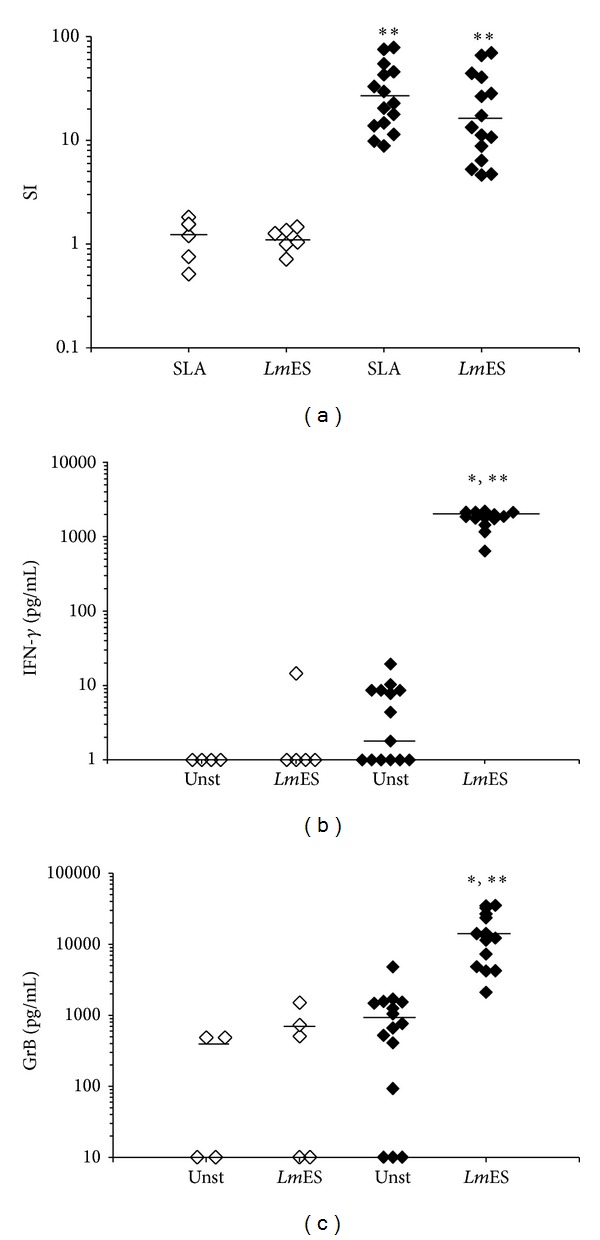
PBMCs response induced by* Lm*ES. PBMCs from HZCL and HC were stimulated with* Lm*ES (10 *μ*g/mL) or SLA (10 *μ*g/mL) during 5 days. (a) Proliferation was assessed by [^3^H]-thymidine uptake. Results were expressed as an index of proliferation (SI) (mean counts of triplicates in antigen-stimulated cultures/mean counts of triplicates in unstimulated cultures) in all tested individuals. IFN-*γ* (b) and GrB (c) levels were evaluated in supernatants of cultures by ELISA tests at day 5. Results were expressed as cytokine concentrations in pg/mL. Bars represent median values. Statistical analysis: *P < 0.05, when comparing the stimulated cells to the unstimulated cells (Unst) and **P < 0.05, when comparing HZCL to HC.

The effect of* Leishmania* proteins on IFN-*γ* and GrB production by immune cells was studied in 15 HZCL individuals in whom PBMCs proliferate when stimulated with* Leishmania *antigens (*Lm*ES and SLA) and 6 HC who do not show any PBMC cell proliferation (Figure 1(a)).* Leishmania *antigens induced significantly high levels of IFN-*γ* and GrB in culture supernatants of PBMCs from HZCL individuals compared to those of HC (*P* < 0.001 and *P* = 0.009, resp.), (Figures 1(b) and 1(c)). Interestingly, a highly significant correlation was found between the levels of GrB and IFN-*γ* produced by* Leishmania* antigen-stimulated PBMCs (Spearman's rank correlation coefficient; *r* = 0.816, *P* < 0.0001), (*data not shown*).

### 3.2. GrB Induced by Leishmania Antigens Are Unexpectedly Produced by CD4^+^ T Cells

**Figure 2:**
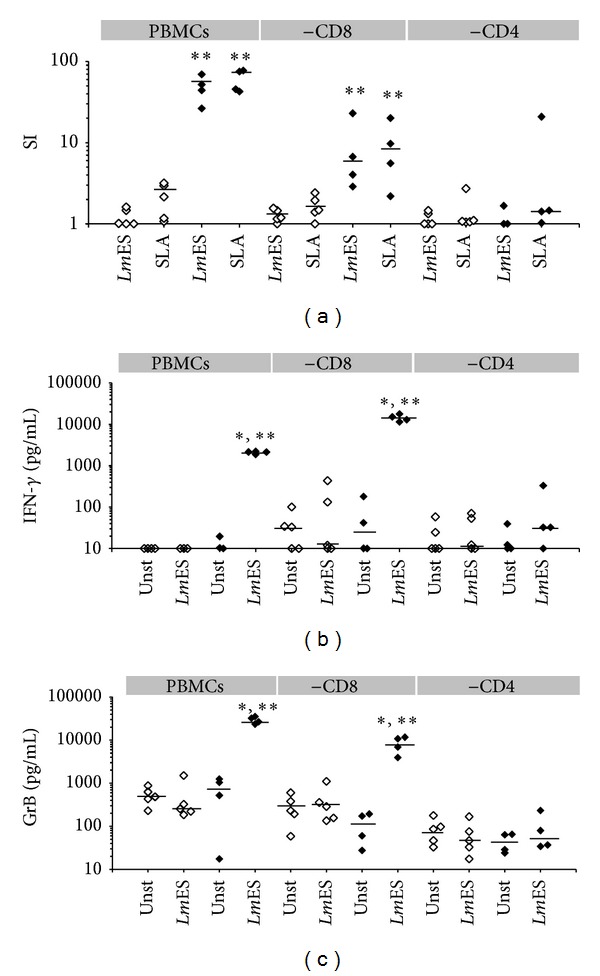
Immune response induced by* Lm*ES in total PBMCs or PBMCs depleted of CD4^+^ or CD8^+^ T cells. Whole PBMCs or those depleted of CD4^+^ (-CD4) or CD8^+^ T cells (-CD8) from 4 HZCL (*◆*) and 5 HC (◊) were stimulated with* Lm*ES (10 *μ*g/mL) for 5 days. (a) Proliferative responses were assessed by [^3^H]-thymidine uptake. IFN-*γ* (b) and GrB (c) secretion was evaluated in supernatants of cultures at day 5 using ELISA tests. Bars represent median values. Statistical analysis: *P < 0.05, when comparing the stimulated cells to the unstimulated cells, (Unst) and **P < 0.05, when comparing HZCL to HC.

In an attempt to better define the features of the cellular population activated by* Leishmania* antigens and more specifically, to determine the effector cells producing IFN-*γ* and GrB, cell depletion experiments were done using PBMCs obtained from four representative HZCL individuals and five donors from the HC group. We first demonstrated that only CD8-depleted PBMCs (-CD8) from HZCL individuals proliferate in response to* Leishmania *antigens (Figure 2(a)). Accordingly, no significant proliferation of CD4-depleted PBMCs (-CD4) was observed in response to stimulation with such antigens. In addition, only total PBMCs or (-CD8) from HZCL individuals produced high levels of IFN-*γ* and GrB in response to* Leishmania *antigen stimulation (Figures 2(b) and 2(c)). In contrast, for the (-CD4), GrB and IFN-*γ* were detected at low levels and no significant differences were observed between unstimulated and stimulated conditions (*P* > 0.05). Altogether, our results suggest that in response to* Leishmania *antigens, the main effector cells were CD4^+^ T cells in term of cell proliferation and IFN-*γ* and GrB production.

### 3.3. IFN-*γ* and GrB Are Produced by Different CD4^+^ T Cell Subset

**Figure 3:**
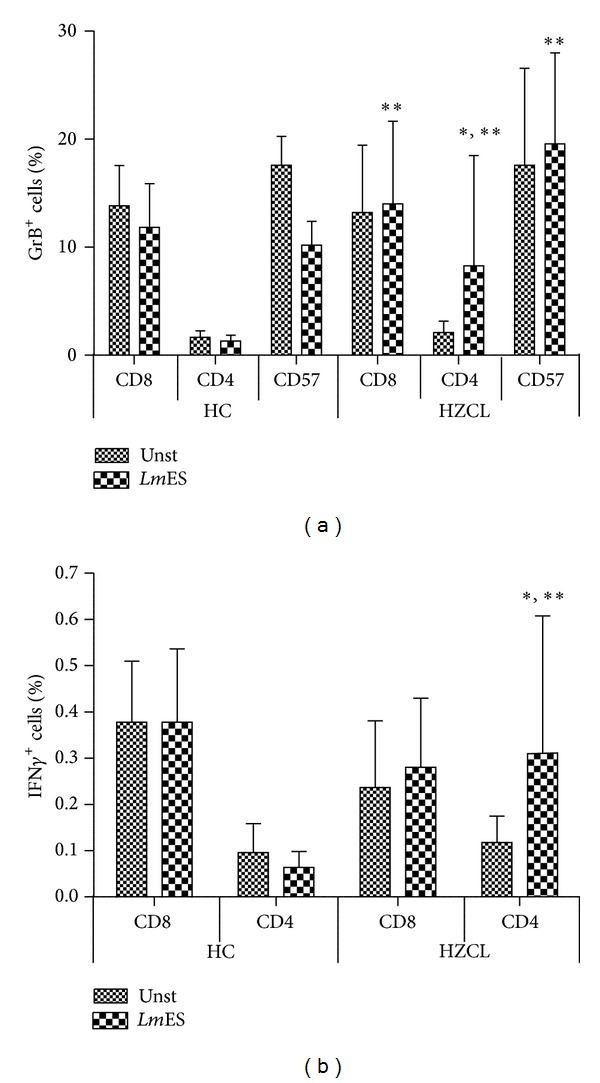
Phenotyping of cells producing GrB and IFN-*γ*. Percentage of cells positive for GrB (a) or IFN-*γ* (b) analyzed* ex-vivo* in PBMCs of HZCL or HC. Results are shown as percentages of positive cells inside the CD3^+^ T cells gate. Results are expressed as the mean ± standard error of the mean of levels. Statistical analysis: *P < 0.05, when comparing the stimulated cells to the unstimulated cells (Unst) and **P < 0.05, when comparing HZCL to HC.

**Figure 4:**
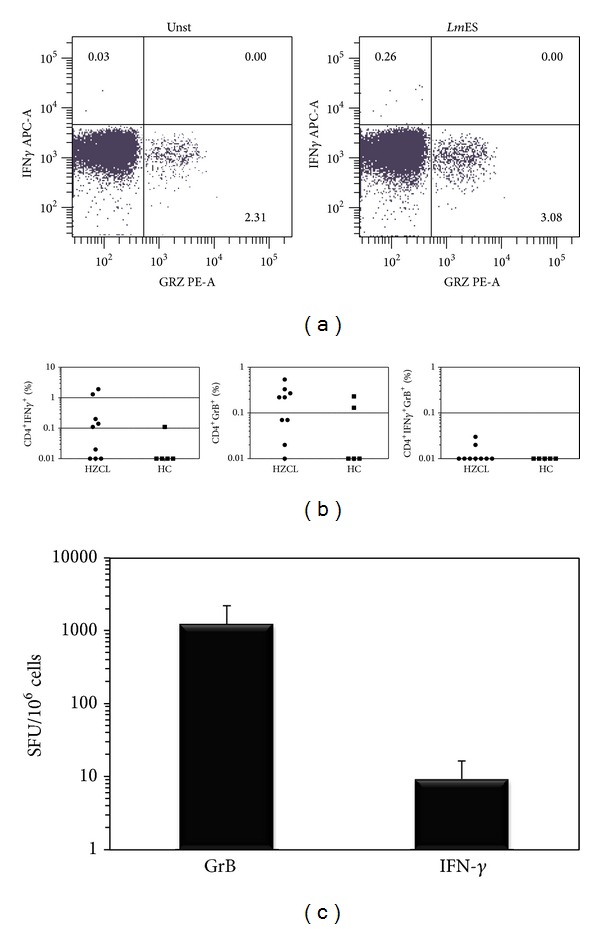
GrB and IFN-*γ* are produced by different subsets of CD4^+^ T cells. (a) Representative example of FACS analyses of PBMCs from HZCL incubated in culture medium alone (Unst) or in presence of proteins (*Lm*ES) and analyzed for the expression of GrB and IFN-*γ*. Results are shown as percentages of positive cells inside the CD4^+^ T cells gate. (b) shows percentages of CD4^+^ T cells positive for GrB, IFN-*γ* and those double positive in 9 HZCL and 5 HC. Results are expressed as the mean ± standard error of the mean of levels. (c) CD4^+^ T cells were purified from the peripheral blood of 4 HZCL incubated with* Lm*ES* in vitro* in presence of mitomycin C-treated autologous PBMCs (APCs) and analysed by a dual color ELISpot specific for GrB and IFN-*γ*. Results are expressed as the mean ± standard error of the mean of SFU/10^6^ cells.

In order to assess if GrB and IFN-*γ* are produced by the same populations of CD4^+^ T cells, intracellular production of IFN-*γ* and GrB in stimulated PBMCs was studied. Flow cytometry analyses performed in 7 HZCL individuals confirmed that stimulation of PBMCs with* Leishmania *antigen proteins induces the production of IFN-*γ* and GrB by CD4^+^ T lymphocytes in most HZCL individuals (Figure 3). In fact, a significant high percentage of CD4^+^ T cells positive for GrB was obtained following* Lm*ES stimulation when compared to that of the unstimulated cells (*P* = 0.044). A similar result was found with regard to the percentage of CD4^+^ T cells positive for IFN-*γ* in PBMCs from HZCL individuals stimulated with* Lm*ES (*P* < 0.0001). Although a high percentage of CD8^+^ T cells and CD57^+^ cells (NKT cells) positive for GrB was found in HZCL individuals compared to that of HC, this percentage did not augment after* Lm*ES stimulation. Similarly, a high percentage of CD4^+^ T cells positive for GrB or IFN-*γ* was found in response to* Lm*ES stimulation in HZCL individuals, yet the absence of a double positive staining of CD4^+^ T cells demonstrated by flow cytometry suggests that both molecules are produced by different CD4^+^ T cell subsets (Figures 4(a) and 4(b)). To confirm these results and for further characterization of this response, we used the Dual color ELISpot assay. CD4^+^ T cells were isolated from PBMCs obtained from four HZCL individuals, challenged with* LmES*, and tested separately for IFN-*γ* and GrB production. Mitomycin C-treated PBMCs were used as APCs. In response to* Lm*ES stimulation, the mean number of GrB positive spots was 1219.91 (±DS = 979.81) SFU/10^6^ CD4^+^ T cells and 9.26 (±DS = 7.09) SFU/10^6^ CD4^+^ T cells for IFN-*γ* (Figure 4(c)). Interestingly, no double positive IFN-*γ* ^+^GrB^+^ spots were detected.

### 3.4. CD4^+^CD25^+^ and CD4^+^CD25^−^ T Cells Produce IFN-*γ* and GrB in Response to LmES

**Figure 5:**
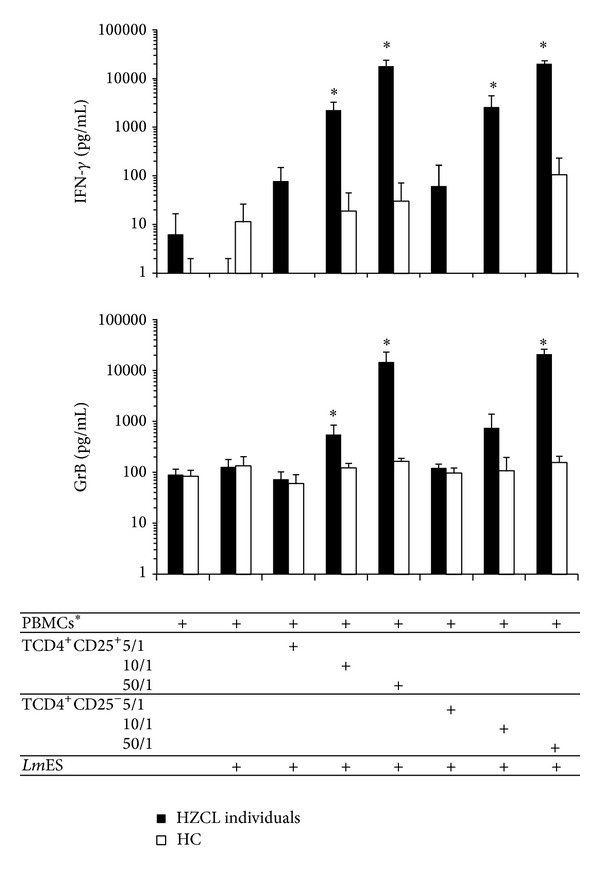
Both CD4^+^CD25^+^ and CD4^+^CD25^−^ T cell subsets produce IFN-*γ* and GrB in response to* Lm*ES* in vitro*. T cell subsets CD4^+^CD25^+^ and CD4^+^CD25^−^ were purified from the peripheral blood of 4 HZCL and 3 HC. The cells were stimulated with* Lm*ES* in vitro* in presence of mitomycin C-treated autologous PBMCs (APCs) at different Effector/APC ratios for 5 days. Culture supernatants were used to determine IFN-*γ* (a) and GrB (b) concentrations using ELISA tests. Results are expressed as the mean ± standard error of the mean of levels. Statistical analysis: *P < 0.05 when comparing HZCL to HC.

Considering the fact that CD4^+^CD25^+^ Treg cells might have cytolytic effects on target T cells, as well as on APCs through the secretion of GrB and perforin [20], we attempted to track their involvement in the production of GrB induced by* Leishmania* antigen stimulation. Therefore, the CD4^+^CD25^+^ and CD4^+^CD25^−^ T cells from 4 HZCL individuals and 3 HC were immunomagnetically purified and cultured with autologous PBMCs treated with mitomycin C (APCs) at different ratios in presence of* Lm*ES, then GrB and IFN-*γ* production was evaluated. As shown in Figure 5, both CD4^+^CD25^+^ and CD4^+^CD25^−^ T subsets from immune individuals produced IFN-*γ* and GrB after* Lm*ES stimulation. IFN-*γ* and GrB levels were enhanced in parallel to the increase of effector cell/APC ratio and reached the highest levels at the ratio of 50/1. At this ratio, IFN-*γ* generated by both T cell populations from HZCL individuals was significantly higher than in HC subjects (*P* = 0.028 and *P* = 0.024 for CD4^+^CD25^+^ and CD4^+^CD25^−^ T cells, resp.). Likewise, significant differences were obtained for GrB levels when comparing HZCL individuals to HC (*P* = 0.034 and *P* = 0.034 for CD4^+^CD25^+^ and CD4^+^CD25^−^ T cells, resp.).

**Figure 6:**
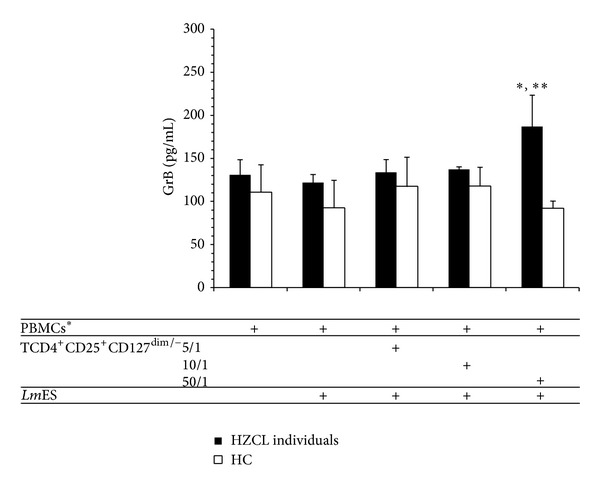
*Lm*ES induce GrB production by CD4^+^CD25^+^CD127^dim/−^ regulatory T in HZCL. CD4^+^CD25^+^CD127^dim/−^ regulatory T cells were purified from peripheral blood of 4 HZCL and 3 HC and stimulated with* Lm*ES (10 *μ*g/mL)* in vitro* in the presence of mitomycin C-treated PBMCs (APCs) at different Effector/APC ratios for 5 days. Culture supernatants were used to determine GrB concentration. Results are expressed as the mean ± standard error of the mean of levels. Statistical analysis: *P < 0.05 when comparing HZCL to HC.

To test whether CD4^+^CD25^+^ T cells activated by* Lm*ES correspond to regulatory T cells, CD4^+^CD25^+^CD127^dim/−^ peripheral regulatory T cells were isolated from cryopreserved cells from the same donors and used in a similar experiment. No IFN-*γ* was detected in supernatants of CD4^+^CD25^+^CD127^dim/−^ peripheral regulatory T cells in all tested conditions (*data not shown*). In contrast, GrB was detected in Treg cell culture supernatants, yet with low levels comparing to the previous experiment, and a significant difference was found between unstimulated and stimulated conditions at the ratio of 50/1 (*P* = 0.024) (Figure 6).

Taken together, our data suggest that stimulation by* Leishmania* antigens activates a CD4^+^CD25^+^ regulatory T cell population that produces GrB.

## 4. Discussion

The rationale for* Leishmania* vaccine development is provided by the evidence that most individuals that had leishmaniasis or symptomless infection are resistant to subsequent symptomatic infections. However, no effective vaccine for human use is currently available. This is mostly due to the difficulties in defining the immunopathological and protective mechanisms in* Leishmania* infections, the nature of effector cells involved in the resistance, and the parasite antigens targeted by this immune response.

In the present study, we aimed at investigating the potential existence of cytotoxic immune response through GrB production against* Leishmania* antigens* ex vivo*, using cells from individuals with a confirmed previous contact with the parasite.

In addition to* Leishmania* soluble antigens, we have chosen to use* Leishmania* excreted/secreted antigens as previous reports have shown that such proteins, released by the parasite in the phagolysosomal compartment, may constitute interesting targets for the host cellular Th1 and cytotoxic immune responses and have been shown to be strongly immunogenic and protective in mice and dogs [12–17]. In humans, few reports have described the cellular immunity against* Leishmania* excreted/secreted proteins [14].

Herein, we report two important findings. The first is that* Leishmania* excreted/secreted antigens are able to induce a similar cellular immune response to that induced by SLA, in terms of intensity of proliferation and cytokine production. Indeed, as these antigens are released into the host cell phagosomes, they might be processed to generate both MHC class II and class I binding peptides and be presented to effector cells. Our results showed evidence of immunogenicity of these proteins in humans, corroborating the previously published data in different experimental models [12–17], and suggest that they are target of a cellular immune response.

Our second finding is that GrB is significantly and specifically produced in response to* Leishmania* antigens in immune individuals. However, unexpectedly, the main source of GrB seems to be CD4^+^ T cells and not CD8^+^ T cells as usually described. In fact, depletion experiments showed that CD4^+^ are as expected the main source of IFN-*γ*, but they also produce high levels of GrB in response to* Leishmania* antigens. Interestingly, IFN-*γ* and GrB production in culture supernatants of stimulated cells from such individuals showed a strong positive correlation (*r* = 0.8), though the absence of double positive IFN-*γ* ^+^GrB^+^ cells in ELISpot and flow cytometry analyses showed that GrB and IFN-*γ* are produced by different CD4^+^ T cell subsets.

Flow cytometry analyses also showed high percentages of CD8^+^ T and CD57^+^ T cells (NKT cells) positive for GrB in HZCL individuals compared with those of HC, but it appears that this increase in percentages is independent of the antigen stimulation and no difference was observed between* Lm*ES-stimulated and -unstimulated PBMCs. Nevertheless, the percentage of CD4^+^GrB^+^ T cells is significantly increased specifically in response to our antigens and these percentages are higher in HZCL individuals compared to those of HC.

To better define these effector cells and check whether regulatory T cells are the source of GrB, additional analyses were performed on CD4^+^ T cells. In fact, studies using mice and human cells suggest that a subset of CD4^+^CD25^+^ T cells can have cytolytic activity. CD4^+^CD25^+^ T cells isolated from human subjects have been shown to express perforin and GrB and exhibit cytotoxicity against a variety of autologous target cells including CD4^+^ and CD8^+^ T cells, CD14^+^ monocytes, and dendritic cells [21]. Our results showed that purified CD4^+^CD25^+^ and CD4^+^CD25^−^ T cell subsets were both able to produce GrB and IFN-*γ* at high levels in HZCL individuals compared to HC. Finally, in order to discriminate between regulatory and activated T cells, CD4^+^CD25^+^CD127^dim/−^ peripheral regulatory T cells were purified. Our data showed their capacity to produce GrB but not IFN-*γ* in response to* Leishmania* antigens in HZCL individuals. Yet, low levels of GrB were detected in culture supernatant of Treg cells. This could be explained by the use of lower numbers of cells after cell sorting from cryopreserved cells. Altogether, our findings may suggest that regulatory T cells are involved, among other cell populations, in GrB production in response to* Leishmania* antigens, suggesting a potential role of a cytotoxic pathway in Treg cell immune regulation in leishmaniasis. Further experiments are in progress to confirm such hypothesis.

Besides their cytotoxic activity, Treg cells have been reported to use several mechanisms to suppress the activation and proliferation of conventional T cells [22]. They can modulate the functions of APCs or proceed through the secretion of inhibitory cytokines, such as TGF-*β*, IL-10, and IL-35 [22]. These mechanisms have not been addressed in the present study. In human cutaneous leishmaniasis, there is a strong correlation between activation of different T cell subsets and disease outcome. The Th1 response is characterized by secretion of proinflammatory cytokines, while Th2 response is associated with anti-inflammatory cytokines, such as IL-10 and TGF-*β*, which promote macrophage inactivation and prevent excessive production of protective cytokines [1]. The role of regulatory T cells in cutaneous leishmaniasis has only recently been investigated. Several groups have shown that during* L. major* infection, CD4^+^CD25^+^ Treg cells accumulate at the primary infection site in both humans and mice where they suppress parasite elimination by CD4^+^CD25^−^ effector cells and mediate chronicity, and their depletion leads to parasite clearance [23–25]. The balance between Th1, Th2, Treg, and other effector cells will determine the disease outcome.

In the present study, while we expected a classical cytotoxic immune response where the main effectors are CD8^+^ T cells, our results surprisingly showed that CD4^+^ T cell populations, including Treg cells, are rather the ones involved in the immune response against* Leishmania* antigens. These CD4^+^ T cells are different from the IFN-*γ*-producing Th1 cells.

Cytotoxic CD4^+^ T cells have been described in several studies and have been implicated in the control of a variety of persistent viral infections, such as EBV, HCV, and HIV-1 infections [26]. Analyses of cytotoxic CD4^+^ T cells indicate that they have lytic granules containing cytotoxic factors, such as granzymes and perforin, and are characterized by a loss of CD28 surface expression [26]. The involvement of such CD28^−^ cytotoxic T lymphocytes in* Leishmania* infection is currently under investigation in our laboratory.

## 5. Conclusion

Our study provides new insights and strong evidence regarding the involvement of CD4^+^ T cells producing GrB in the immune response against* Leishmania* antigens. This cell population deserves further consideration during* Leishmania* infection to figure out whether they are actually involved in the promotion of or protection against leishmaniasis development.

## Figures and Tables

**Table 1:** Clinical and immunological features of the study population.

	HZCL Individuals (*n* = 15)	HC (*n* = 13)
Age, mean ± SD (range), years	37.6 ± 12.9 (15–52)	27.6 ± 3.6 (22–33)
ZCL scars, yes/no	11/4	0/13
Male/female	6/9	2/11
LST induration, mean ± SD (range), mm	10.5 ± 4.6 (5–17.5)	Not available
LST positive^a^	12/15	Not available
SLA-specific lymphoproliferation (IS)		
mean ± SD (range)	31.8 ± 22 (8.8–78.2)	2.2 ± 0.6 (0.5–2.9)

ZCL: zoonotic cutaneous leishmaniasis; LST: leishmanin skin test; IS: index of stimulation.

^a^Number of individuals with a positive LST result/total number of tested individuals.

## References

[1] Reithinger R, Dujardin JC, Louzir H, Pirmez C, Alexander B, Brooker S (2007). Cutaneous leishmaniasis. *Lancet Infectious Diseases*.

[2] Ashford RW (2000). The leishmaniases as emerging and reemerging zoonoses. *International Journal for Parasitology*.

[3] Liew FY, O'Donnell CA (1993). Immunology of leishmaniasis. *Advances in Parasitology*.

[4] Guirges SY (1971). Natural and experimental re-infection of man with Oriental sore. *Annals of Tropical Medicine and Parasitology*.

[5] Davies CR, Llanos-Cuentas EA, Pyke SDM, Dye C (1995). Cutaneous leishmaniasis in the Peruvian Andes: an epidemiological study of infection and immunity. *Epidemiology and Infection*.

[6] Bousoffara T, Louzir H, Ben Salah A, Dellagi K (2004). Analysis of granzyme B activity as a surrogate marker of Leishmania-specific cell-mediated cytotoxicity in zoonotic cutaneous leishmaniasis. *Journal of Infectious Diseases*.

[7] Louzir H, Bousoffara T, Ben Salah A Leishmania specific cytotoxic cellular immune response as a new correlate for human protection against infection.

[8] Santarém N, Silvestre R, Tavares J (2007). Immune response regulation by *Leishmania* secreted and non-secreted antigens. *Journal of Biomedicine and Biotechnology*.

[9] Costa-Silva TA, Meira CS, Ferreira IMR, Hiramoto RM, Pereira-Chioccola VL (2008). Evaluation of immunization with tachyzoite excreted-secreted proteins in a novel susceptible mouse model (A/Sn) for Toxoplasma gondii. *Experimental Parasitology*.

[10] Grotzke JE, Siler AC, Lewinsohn DA, Lewinsohn DM (2010). Secreted immunodominant *Mycobacterium tuberculosis* antigens are processed by the cytosolic pathway. *Journal of Immunology*.

[11] Lv H, Gao Y, Wu Y (2010). Identification of a novel cytotoxic T lymphocyte epitope from CFP21, a secreted protein of *Mycobacterium tuberculosis*. *Immunology Letters*.

[12] Rosa R, Rodrigues OR, Marques C, Santos-Gomes GM (2005). *Leishmania infantum*: soluble proteins released by the parasite exert differential effects on host immune response. *Experimental Parasitology*.

[13] Rosa R, Marques C, Rodrigues OR, Santos-Gomes GM (2007). Immunization with *Leishmania infantum* released proteins confers partial protection against parasite infection with a predominant Th1 specific immune response. *Vaccine*.

[14] Tonui WK, Titus RG (2006). *Leishmania major* soluble exo-antigens (*Lm*SEAgs) protect neonatal BALB/c mice from a subsequent challenge with *L. major* and stimulate cytokine production by Leishmania-naïve human peripheral blood mononuclear cells. *Journal of Parasitology*.

[15] Lemesre J, Holzmuller P, Cavaleyra M, Gonçalves RB, Hottin G, Papierok G (2005). Protection against experimental visceral leishmaniasis infection in dogs immunized with purified excreted secreted antigens of *Leishmania infantum* promastigotes. *Vaccine*.

[16] Lemesre J, Holzmuller P, Gonçalves RB (2007). Long-lasting protection against canine visceral leishmaniasis using the LiESAp-MDP vaccine in endemic areas of France: double-blind randomised efficacy field trial. *Vaccine*.

[17] Holzmuller P, Cavaleyra M, Moreaux J (2005). Lymphocytes of dogs immunised with purified excreted-secreted antigens of *Leishmania infantum* co-incubated with *Leishmania* infected macrophages produce IFN gamma resulting in nitric oxide-mediated amastigote apoptosis. *Veterinary Immunology and Immunopathology*.

[18] Sassi A, Louzir H, Salah AB, Mokni M, Osman AB, Dellagi K (1999). Leishmanin skin test lymphoproliferative responses and cytokine production after symptomatic or asymptomatic *Leishmania major* infection in Tunisia. *Clinical and Experimental Immunology*.

[19] Chenik M, Lakhal S, Ben Khalef N, Zribi L, Louzir H, Dellagi K (2006). Approaches for the identification of potential excreted/secreted proteins of *Leishmania major* parasites. *Parasitology*.

[20] Rochman Y, Spolski R, Leonard WJ (2009). New insights into the regulation of T cells by *γ*
_c_ family cytokines. *Nature Reviews Immunology*.

[21] Grossman WJ, Verbsky JW, Barchet W, Colonna M, Atkinson JP, Ley TJ (2004). Human T regulatory cells can use the perforin pathway to cause autologous target cell death. *Immunity*.

[22] Vignali DAA, Collison LW, Workman CJ (2008). How regulatory T cells work. *Nature Reviews Immunology*.

[23] Anderson CF, Mendez S, Sacks DL (2005). Nonhealing infection despite Th1 polarization produced by a strain of *Leishmania* major in C57BL/6 mice. *Journal of Immunology*.

[24] Campanelli AP, Roselino AM, Cavassani KA (2006). CD4^+^CD25^+^ T cells in skin lesions of patients with cutaneous leishmaniasis exhibit phenotypic and functional characteristics of natural regulatory T cells. *Journal of Infectious Diseases*.

[25] Bourreau E, Ronet C, Darcissac E (2009). Intralesional regulatory T-Cell suppressive function during human acute and chronic cutaneous leishmaniasis due to *Leishmania guyanensis*. *Infection and Immunity*.

[26] Soghoian DZ, Streeck H (2010). Cytolytic CD4^+^ T cells in viral immunity. *Expert Review of Vaccines*.

